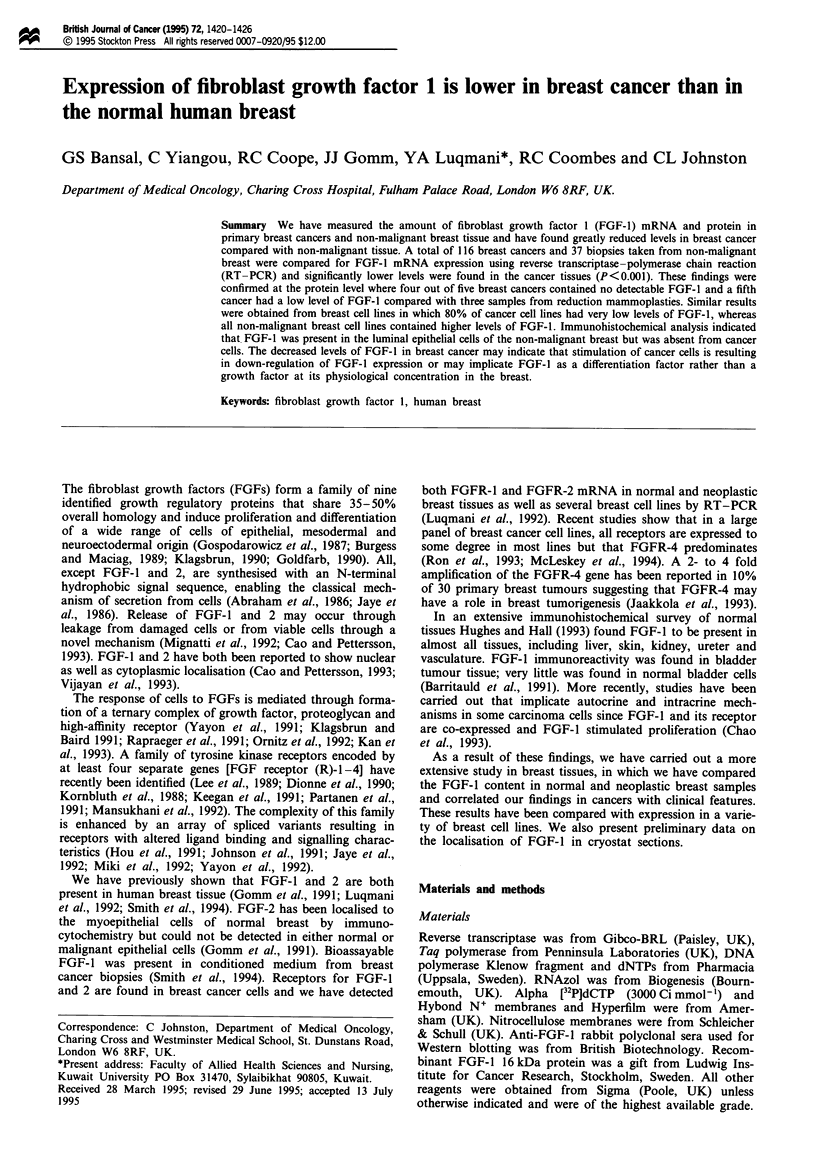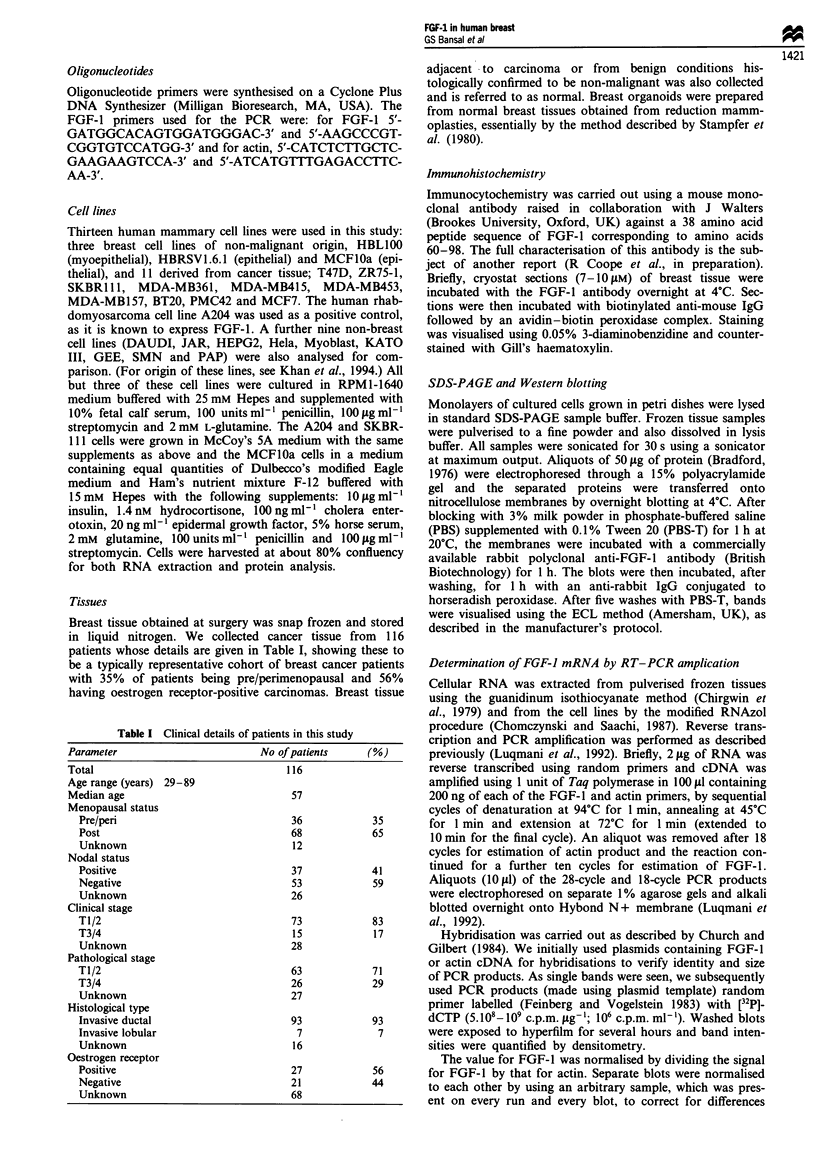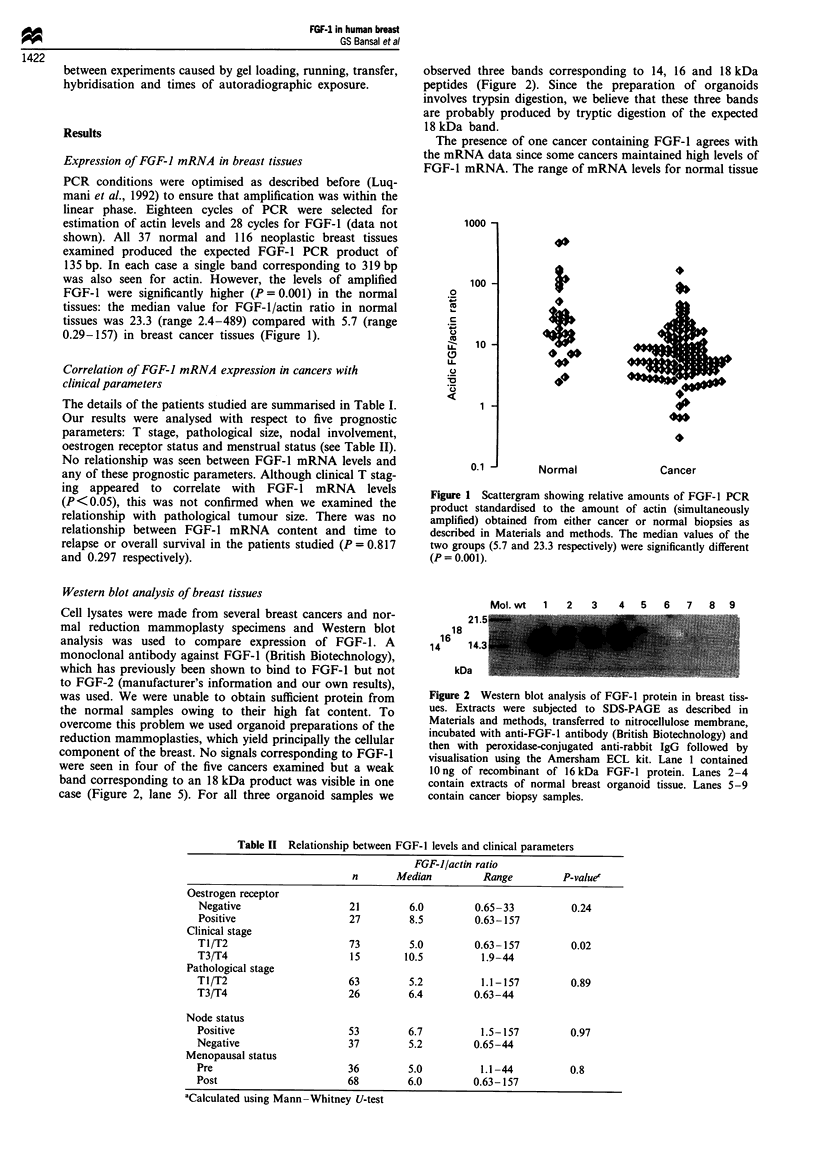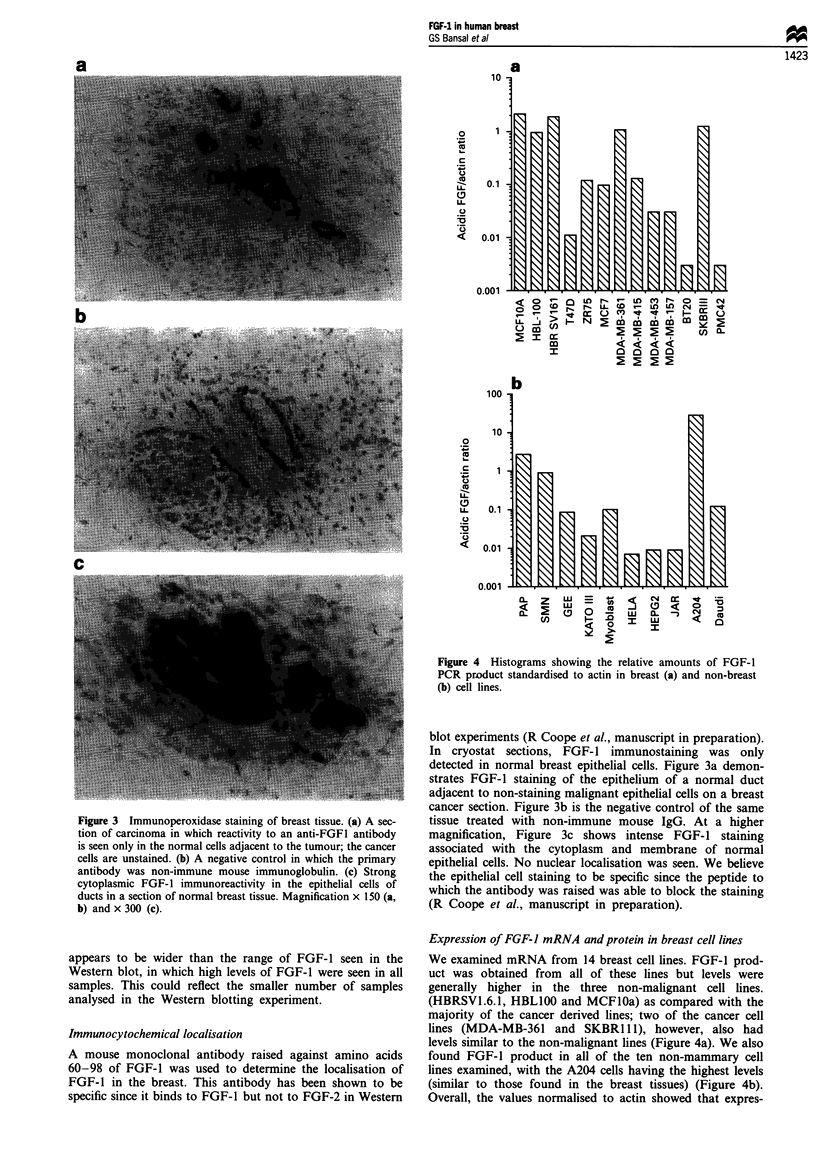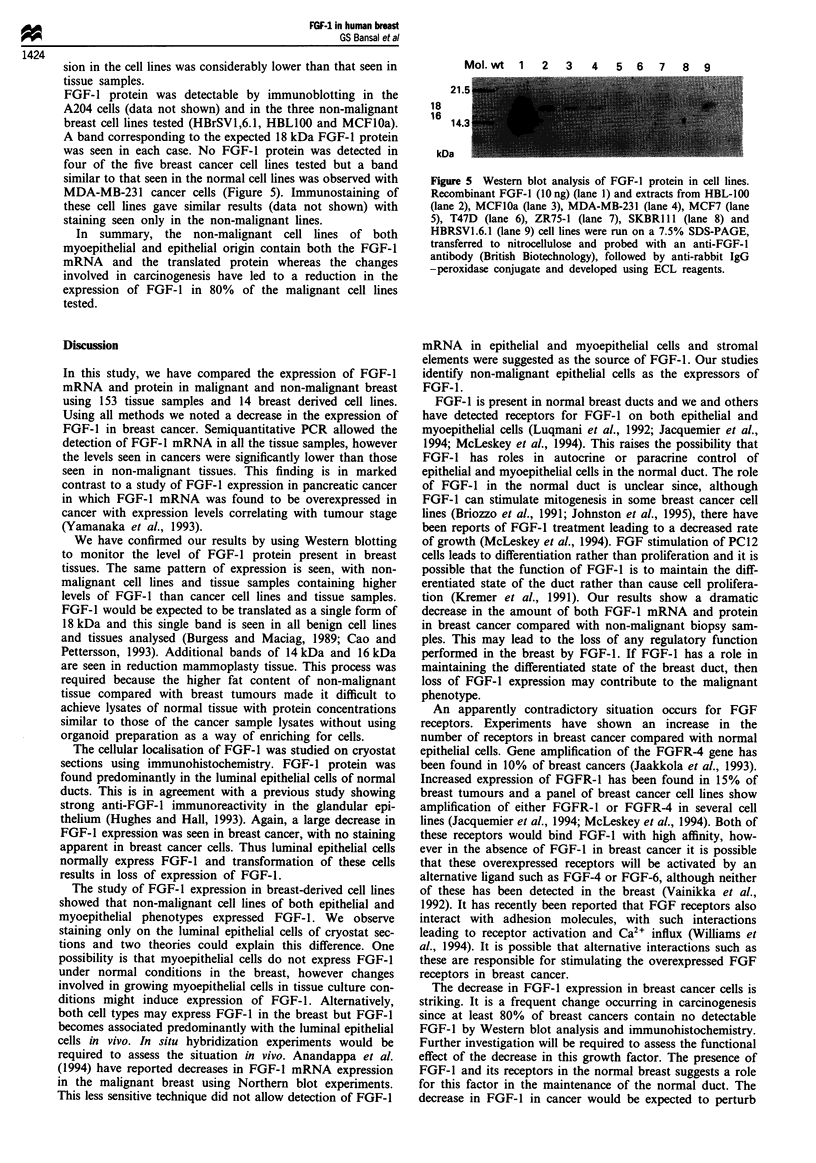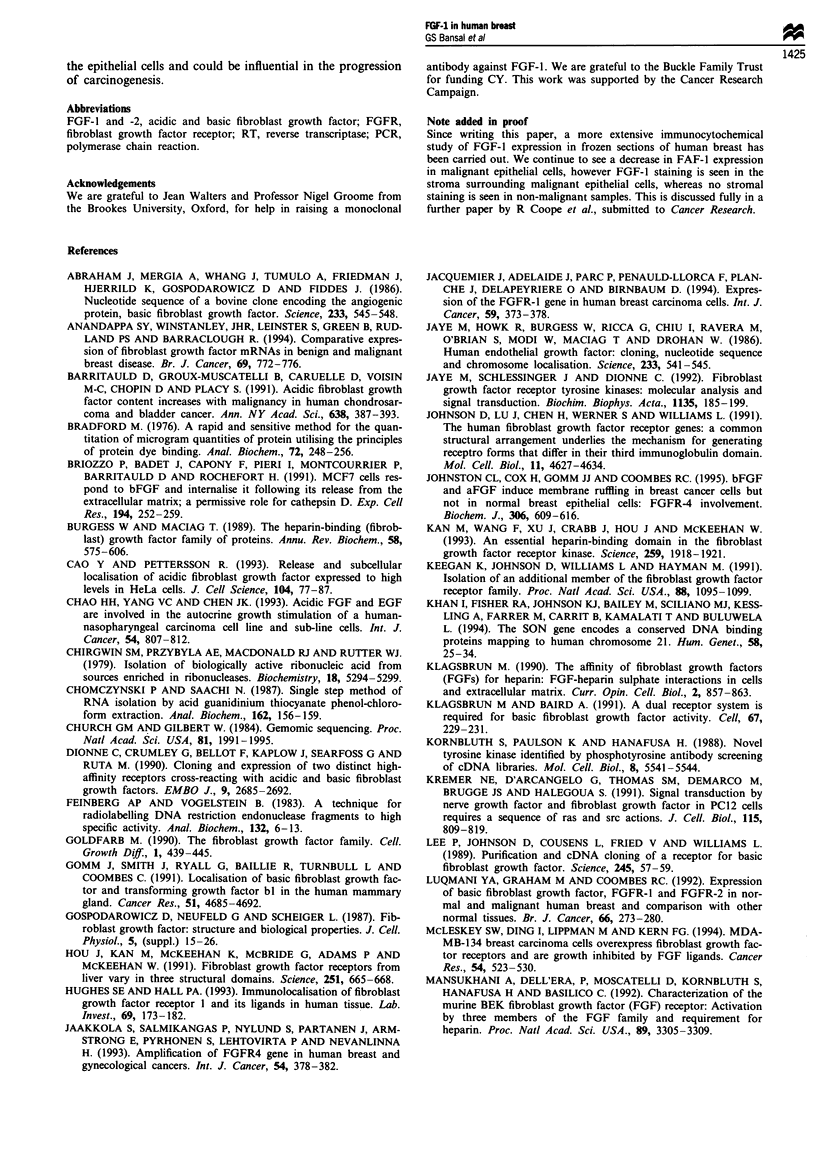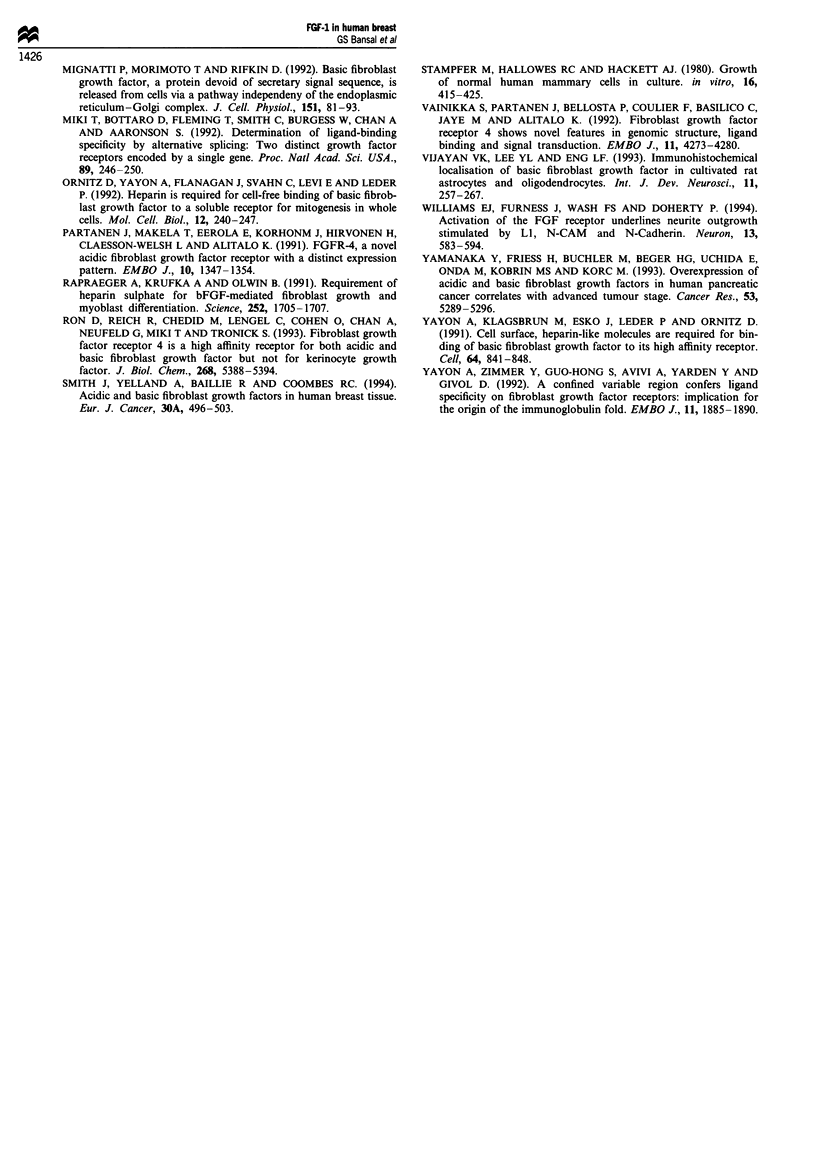# Expression of fibroblast growth factor 1 is lower in breast cancer than in the normal human breast.

**DOI:** 10.1038/bjc.1995.524

**Published:** 1995-12

**Authors:** G. S. Bansal, C. Yiangou, R. C. Coope, J. J. Gomm, Y. A. Luqmani, R. C. Coombes, C. L. Johnston

**Affiliations:** Department of Medical Oncology, Charing Cross Hospital, London, UK.

## Abstract

**Images:**


British Journal of Cancer (1995) 72, 1420–1426
© 1995 Stockton Press All rights reserved 0007–0920/95 $12.00

### Expression of fibroblast growth factor 1 is lower in breast cancer than in the normal human breast

GS Bansal, C Yiangou, RC Coope, JJ Gomm, YA Luqmani*, RC Coombes and CL Johnston

*Department of Medical Oncology, Charing Cross Hospital, Fulham Palace Road, London W6 8RF, UK.*

**Summary** We have measured the amount of fibroblast growth factor 1 (FGF-1) mRNA and protein in primary breast cancers and non-malignant breast tissue and have found greatly reduced levels in breast cancer compared with non-malignant tissue. A total of 116 breast cancers and 37 biopsies taken from non-malignant breast were compared for FGF-1 mRNA expression using reverse transcriptase–polymerase chain reaction (RT–PCR) and significantly lower levels were found in the cancer tissues ($P<0.001$). These findings were confirmed at the protein level where four out of five breast cancers contained no detectable FGF-1 and a fifth cancer had a low level of FGF-1 compared with three samples from reduction mammoplasties. Similar results were obtained from breast cell lines in which 80% of cancer cell lines had very low levels of FGF-1, whereas all non-malignant breast cell lines contained higher levels of FGF-1. Immunohistochemical analysis indicated that FGF-1 was present in the luminal epithelial cells of the non-malignant breast but was absent from cancer cells. The decreased levels of FGF-1 in breast cancer may indicate that stimulation of cancer cells is resulting in down-regulation of FGF-1 expression or may implicate FGF-1 as a differentiation factor rather than a growth factor at its physiological concentration in the breast.

**Keywords:** fibroblast growth factor 1, human breast

The fibroblast growth factors (FGFs) form a family of nine identified growth regulatory proteins that share 35–50% overall homology and induce proliferation and differentiation of a wide range of cells of epithelial, mesodermal and neuroectodermal origin (Gospodarowicz *et al.*, 1987; Burgess and Maciag, 1989; Klagsbrun, 1990; Goldfarb, 1990). All, except FGF-1 and 2, are synthesised with an N-terminal hydrophobic signal sequence, enabling the classical mechanism of secretion from cells (Abraham *et al.*, 1986; Jaye *et al.*, 1986). Release of FGF-1 and 2 may occur through leakage from damaged cells or from viable cells through a novel mechanism (Mignatti *et al.*, 1992; Cao and Pettersson, 1993). FGF-1 and 2 have both been reported to show nuclear as well as cytoplasmic localisation (Cao and Pettersson, 1993; Vijayan *et al.*, 1993).

The response of cells to FGFs is mediated through formation of a ternary complex of growth factor, proteoglycan and high-affinity receptor (Yayon *et al.*, 1991; Klagsbrun and Baird 1991; Rapraeger *et al.*, 1991; Ornitz *et al.*, 1992; Kan *et al.*, 1993). A family of tyrosine kinase receptors encoded by at least four separate genes [FGF receptor (R)-1–4] have recently been identified (Lee *et al.*, 1989; Dionne *et al.*, 1990; Kornbluth *et al.*, 1988; Keegan *et al.*, 1991; Partanen *et al.*, 1991; Mansukhani *et al.*, 1992). The complexity of this family is enhanced by an array of spliced variants resulting in receptors with altered ligand binding and signalling characteristics (Hou *et al.*, 1991; Johnson *et al.*, 1991; Jaye *et al.*, 1992; Miki *et al.*, 1992; Yayon *et al.*, 1992).

We have previously shown that FGF-1 and 2 are both present in human breast tissue (Gomm *et al.*, 1991; Luqmani *et al.*, 1992; Smith *et al.*, 1994). FGF-2 has been localised to the myoepithelial cells of normal breast by immunocytochemistry but could not be detected in either normal or malignant epithelial cells (Gomm *et al.*, 1991). Bioassayable FGF-1 was present in conditioned medium from breast cancer biopsies (Smith *et al.*, 1994). Receptors for FGF-1 and 2 are found in breast cancer cells and we have detected both FGFR-1 and FGFR-2 mRNA in normal and neoplastic breast tissues as well as several breast cell lines by RT–PCR (Luqmani *et al.*, 1992). Recent studies show that in a large panel of breast cancer cell lines, all receptors are expressed to some degree in most lines but that FGFR-4 predominates (Ron *et al.*, 1993; McLeskey *et al.*, 1994). A 2- to 4 fold amplification of the FGFR-4 gene has been reported in 10% of 30 primary breast tumours suggesting that FGFR-4 may have a role in breast tumorigenesis (Jaakkola *et al.*, 1993).

In an extensive immunohistochemical survey of normal tissues Hughes and Hall (1993) found FGF-1 to be present in almost all tissues, including liver, skin, kidney, ureter and vasculature. FGF-1 immunoreactivity was found in bladder tumour tissue; very little was found in normal bladder cells (Barritauld *et al.*, 1991). More recently, studies have been carried out that implicate autocrine and intracrine mechanisms in some carcinoma cells since FGF-1 and its receptor are co-expressed and FGF-1 stimulated proliferation (Chao *et al.*, 1993).

As a result of these findings, we have carried out a more extensive study in breast tissues, in which we have compared the FGF-1 content in normal and neoplastic breast samples and correlated our findings in cancers with clinical features. These results have been compared with expression in a variety of breast cell lines. We also present preliminary data on the localisation of FGF-1 in cryostat sections.

### Materials and methods

### *Materials*

Reverse transcriptase was from Gibco-BRL (Paisley, UK), *Taq* polymerase from Penninsula Laboratories (UK), DNA polymerase Klenow fragment and dNTPs from Pharmacia (Uppsala, Sweden). RNAzol was from Biogenesis (Bournemouth, UK). Alpha [$^{32}$P]dCTP (3000 Ci mmol$^{-1}$) and Hybond N$^+$ membranes and Hyperfilm were from Amersham (UK). Nitrocellulose membranes were from Schleicher & Schull (UK). Anti-FGF-1 rabbit polyclonal sera used for Western blotting was from British Biotechnology. Recombinant FGF-1 16 kDa protein was a gift from Ludwig Institute for Cancer Research, Stockholm, Sweden. All other reagents were obtained from Sigma (Poole, UK) unless otherwise indicated and were of the highest available grade.

Correspondence: C Johnston, Department of Medical Oncology, Charing Cross and Westminster Medical School, St. Dunstans Road, London W6 8RF, UK.
*Present address: Faculty of Allied Health Sciences and Nursing, Kuwait University PO Box 31470, Sylaibikhat 90805, Kuwait.
Received 28 March 1995; revised 29 June 1995; accepted 13 July 1995

*Oligonucleotides*

Oligonucleotide primers were synthesised on a Cyclone Plus DNA Synthesizer (Milligan Bioresearch, MA, USA). The FGF-1 primers used for the PCR were: for FGF-1 5'-GATGGCACAGTGGATGGGAC-3' and 5'-AAGCCCGT-CGGTGTCCATGG-3' and for actin, 5'-CATCTCTTGCTC-GAAGAAGTCCA-3' and 5'-ATCATGTTTGAGACCTTC-AA-3'.

*Cell lines*

Thirteen human mammary cell lines were used in this study: three breast cell lines of non-malignant origin, HBL100 (myoepithelial), HBRSV1.6.1 (epithelial) and MCF10a (epithelial), and 11 derived from cancer tissue; T47D, ZR75-1, SKBR111, MDA-MB361, MDA-MB415, MDA-MB453, MDA-MB157, BT20, PMC42 and MCF7. The human rhabdomyosarcoma cell line A204 was used as a positive control, as it is known to express FGF-1. A further nine non-breast cell lines (DAUDI, JAR, HEPG2, Hela, Myoblast, KATO III, GEE, SMN and PAP) were also analysed for comparison. (For origin of these lines, see Khan *et al.*, 1994.) All but three of these cell lines were cultured in RPM1-1640 medium buffered with 25 mM Hepes and supplemented with 10% fetal calf serum, 100 units $ml^{-1}$ penicillin, 100 $\mu g\ ml^{-1}$ streptomycin and 2 mM L-glutamine. The A204 and SKBR-111 cells were grown in McCoy's 5A medium with the same supplements as above and the MCF10a cells in a medium containing equal quantities of Dulbecco's modified Eagle medium and Ham's nutrient mixture F-12 buffered with 15 mM Hepes with the following supplements: 10 $\mu g\ ml^{-1}$ insulin, 1.4 nM hydrocortisone, 100 ng $ml^{-1}$ cholera enterotoxin, 20 ng $ml^{-1}$ epidermal growth factor, 5% horse serum, 2 mM glutamine, 100 units $ml^{-1}$ penicillin and 100 $\mu g\ ml^{-1}$ streptomycin. Cells were harvested at about 80% confluency for both RNA extraction and protein analysis.

*Tissues*

Breast tissue obtained at surgery was snap frozen and stored in liquid nitrogen. We collected cancer tissue from 116 patients whose details are given in Table I, showing these to be a typically representative cohort of breast cancer patients with 35% of patients being pre/perimenopausal and 56% having oestrogen receptor-positive carcinomas. Breast tissue adjacent to carcinoma or from benign conditions histologically confirmed to be non-malignant was also collected and is referred to as normal. Breast organoids were prepared from normal breast tissues obtained from reduction mammoplasties, essentially by the method described by Stampfer *et al.* (1980).

**Table I** Clinical details of patients in this study

| *Parameter* | | *No of patients* | *(%)* |
|---|---|---|---|
| Total | | 116 | |
| Age range (years) | 29–89 | | |
| Median age | | 57 | |
| Menopausal status | | | |
| Pre/peri | | 36 | 35 |
| Post | | 68 | 65 |
| Unknown | | 12 | |
| Nodal status | | | |
| Positive | | 37 | 41 |
| Negative | | 53 | 59 |
| Unknown | | 26 | |
| Clinical stage | | | |
| T1/2 | | 73 | 83 |
| T3/4 | | 15 | 17 |
| Unknown | | 28 | |
| Pathological stage | | | |
| T1/2 | | 63 | 71 |
| T3/4 | | 26 | 29 |
| Unknown | | 27 | |
| Histological type | | | |
| Invasive ductal | | 93 | 93 |
| Invasive lobular | | 7 | 7 |
| Unknown | | 16 | |
| Oestrogen receptor | | | |
| Positive | | 27 | 56 |
| Negative | | 21 | 44 |
| Unknown | | 68 | |

*Immunohistochemistry*

Immunocytochemistry was carried out using a mouse monoclonal antibody raised in collaboration with J Walters (Brookes University, Oxford, UK) against a 38 amino acid peptide sequence of FGF-1 corresponding to amino acids 60–98. The full characterisation of this antibody is the subject of another report (R Coope *et al.*, in preparation). Briefly, cryostat sections (7–10 $\mu M$) of breast tissue were incubated with the FGF-1 antibody overnight at 4°C. Sections were then incubated with biotinylated anti-mouse IgG followed by an avidin–biotin peroxidase complex. Staining was visualised using 0.05% 3-diaminobenzidine and counterstained with Gill's haematoxylin.

*SDS-PAGE and Western blotting*

Monolayers of cultured cells grown in petri dishes were lysed in standard SDS-PAGE sample buffer. Frozen tissue samples were pulverised to a fine powder and also dissolved in lysis buffer. All samples were sonicated for 30 s using a sonicator at maximum output. Aliquots of 50 $\mu g$ of protein (Bradford, 1976) were electrophoresed through a 15% polyacrylamide gel and the separated proteins were transferred onto nitrocellulose membranes by overnight blotting at 4°C. After blocking with 3% milk powder in phosphate-buffered saline (PBS) supplemented with 0.1% Tween 20 (PBS-T) for 1 h at 20°C, the membranes were incubated with a commercially available rabbit polyclonal anti-FGF-1 antibody (British Biotechnology) for 1 h. The blots were then incubated, after washing, for 1 h with an anti-rabbit IgG conjugated to horseradish peroxidase. After five washes with PBS-T, bands were visualised using the ECL method (Amersham, UK), as described in the manufacturer's protocol.

*Determination of FGF-1 mRNA by RT–PCR amplication*

Cellular RNA was extracted from pulverised frozen tissues using the guanidinum isothiocyanate method (Chirgwin *et al.*, 1979) and from the cell lines by the modified RNAzol procedure (Chomczynski and Saachi, 1987). Reverse transcription and PCR amplification was performed as described previously (Luqmani *et al.*, 1992). Briefly, 2 $\mu g$ of RNA was reverse transcribed using random primers and cDNA was amplified using 1 unit of *Taq* polymerase in 100 $\mu l$ containing 200 ng of each of the FGF-1 and actin primers, by sequential cycles of denaturation at 94°C for 1 min, annealing at 45°C for 1 min and extension at 72°C for 1 min (extended to 10 min for the final cycle). An aliquot was removed after 18 cycles for estimation of actin product and the reaction continued for a further ten cycles for estimation of FGF-1. Aliquots (10 $\mu l$) of the 28-cycle and 18-cycle PCR products were electrophoresed on separate 1% agarose gels and alkali blotted overnight onto Hybond N+ membrane (Luqmani *et al.*, 1992).

Hybridisation was carried out as described by Church and Gilbert (1984). We initially used plasmids containing FGF-1 or actin cDNA for hybridisations to verify identity and size of PCR products. As single bands were seen, we subsequently used PCR products (made using plasmid template) random primer labelled (Feinberg and Vogelstein 1983) with [$^{32}$P]-dCTP ($5.10^8$–$10^9$ c.p.m. $\mu g^{-1}$; $10^6$ c.p.m. $ml^{-1}$). Washed blots were exposed to hyperfilm for several hours and band intensities were quantified by densitometry.

The value for FGF-1 was normalised by dividing the signal for FGF-1 by that for actin. Separate blots were normalised to each other by using an arbitrary sample, which was present on every run and every blot, to correct for differences between experiments caused by gel loading, running, transfer, hybridisation and times of autoradiographic exposure.

### Results

### *Expression of FGF-1 mRNA in breast tissues*

PCR conditions were optimised as described before (Luqmani *et al.*, 1992) to ensure that amplification was within the linear phase. Eighteen cycles of PCR were selected for estimation of actin levels and 28 cycles for FGF-1 (data not shown). All 37 normal and 116 neoplastic breast tissues examined produced the expected FGF-1 PCR product of 135 bp. In each case a single band corresponding to 319 bp was also seen for actin. However, the levels of amplified FGF-1 were significantly higher ($P = 0.001$) in the normal tissues: the median value for FGF-1/actin ratio in normal tissues was 23.3 (range 2.4–489) compared with 5.7 (range 0.29–157) in breast cancer tissues (Figure 1).

### *Correlation of FGF-1 mRNA expression in cancers with clinical parameters*

The details of the patients studied are summarised in Table I. Our results were analysed with respect to five prognostic parameters: T stage, pathological size, nodal involvement, oestrogen receptor status and menstrual status (see Table II). No relationship was seen between FGF-1 mRNA levels and any of these prognostic parameters. Although clinical T staging appeared to correlate with FGF-1 mRNA levels ($P < 0.05$), this was not confirmed when we examined the relationship with pathological tumour size. There was no relationship between FGF-1 mRNA content and time to relapse or overall survival in the patients studied ($P = 0.817$ and 0.297 respectively).

### *Western blot analysis of breast tissues*

Cell lysates were made from several breast cancers and normal reduction mammoplasty specimens and Western blot analysis was used to compare expression of FGF-1. A monoclonal antibody against FGF-1 (British Biotechnology), which has previously been shown to bind to FGF-1 but not to FGF-2 (manufacturer's information and our own results), was used. We were unable to obtain sufficient protein from the normal samples owing to their high fat content. To overcome this problem we used organoid preparations of the reduction mammoplasties, which yield principally the cellular component of the breast. No signals corresponding to FGF-1 were seen in four of the five cancers examined but a weak band corresponding to an 18 kDa product was visible in one case (Figure 2, lane 5). For all three organoid samples we observed three bands corresponding to 14, 16 and 18 kDa peptides (Figure 2). Since the preparation of organoids involves trypsin digestion, we believe that these three bands are probably produced by tryptic digestion of the expected 18 kDa band.

The presence of one cancer containing FGF-1 agrees with the mRNA data since some cancers maintained high levels of FGF-1 mRNA. The range of mRNA levels for normal tissue

**Figure 1** Scattergram showing relative amounts of FGF-1 PCR product standardised to the amount of actin (simultaneously amplified) obtained from either cancer or normal biopsies as described in Materials and methods. The median values of the two groups (5.7 and 23.3 respectively) were significantly different ($P = 0.001$).

**Figure 2** Western blot analysis of FGF-1 protein in breast tissues. Extracts were subjected to SDS-PAGE as described in Materials and methods, transferred to nitrocellulose membrane, incubated with anti-FGF-1 antibody (British Biotechnology) and then with peroxidase-conjugated anti-rabbit IgG followed by visualisation using the Amersham ECL kit. Lane 1 contained 10 ng of recombinant of 16 kDa FGF-1 protein. Lanes 2–4 contain extracts of normal breast organoid tissue. Lanes 5–9 contain cancer biopsy samples.

**Table II** Relationship between FGF-1 levels and clinical parameters

| | *n* | *FGF-1/actin ratio* *Median* | *Range* | *P-value*[a] |
|---|---|---|---|---|
| Oestrogen receptor | | | | |
| Negative | 21 | 6.0 | 0.65–33 | 0.24 |
| Positive | 27 | 8.5 | 0.63–157 | |
| Clinical stage | | | | |
| T1/T2 | 73 | 5.0 | 0.63–157 | 0.02 |
| T3/T4 | 15 | 10.5 | 1.9–44 | |
| Pathological stage | | | | |
| T1/T2 | 63 | 5.2 | 1.1–157 | 0.89 |
| T3/T4 | 26 | 6.4 | 0.63–44 | |
| Node status | | | | |
| Positive | 53 | 6.7 | 1.5–157 | 0.97 |
| Negative | 37 | 5.2 | 0.65–44 | |
| Menopausal status | | | | |
| Pre | 36 | 5.0 | 1.1–44 | 0.8 |
| Post | 68 | 6.0 | 0.63–157 | |

[a]Calculated using Mann–Whitney *U*-test

**Figure 3** Immunoperoxidase staining of breast tissue. (**a**) A section of carcinoma in which reactivity to an anti-FGF1 antibody is seen only in the normal cells adjacent to the tumour; the cancer cells are unstained. (**b**) A negative control in which the primary antibody was non-immune mouse immunoglobulin. (**c**) Strong cytoplasmic FGF-1 immunoreactivity in the epithelial cells of ducts in a section of normal breast tissue. Magnification × 150 (**a**, **b**) and × 300 (**c**).

appears to be wider than the range of FGF-1 seen in the Western blot, in which high levels of FGF-1 were seen in all samples. This could reflect the smaller number of samples analysed in the Western blotting experiment.

*Immunocytochemical localisation*

A mouse monoclonal antibody raised against amino acids 60–98 of FGF-1 was used to determine the localisation of FGF-1 in the breast. This antibody has been shown to be specific since it binds to FGF-1 but not to FGF-2 in Western

**Figure 4** Histograms showing the relative amounts of FGF-1 PCR product standardised to actin in breast (**a**) and non-breast (**b**) cell lines.

blot experiments (R Coope *et al.*, manuscript in preparation). In cryostat sections, FGF-1 immunostaining was only detected in normal breast epithelial cells. Figure 3a demonstrates FGF-1 staining of the epithelium of a normal duct adjacent to non-staining malignant epithelial cells on a breast cancer section. Figure 3b is the negative control of the same tissue treated with non-immune mouse IgG. At a higher magnification, Figure 3c shows intense FGF-1 staining associated with the cytoplasm and membrane of normal epithelial cells. No nuclear localisation was seen. We believe the epithelial cell staining to be specific since the peptide to which the antibody was raised was able to block the staining (R Coope *et al.*, manuscript in preparation).

*Expression of FGF-1 mRNA and protein in breast cell lines*

We examined mRNA from 14 breast cell lines. FGF-1 product was obtained from all of these lines but levels were generally higher in the three non-malignant cell lines. (HBRSV1.6.1, HBL100 and MCF10a) as compared with the majority of the cancer derived lines; two of the cancer cell lines (MDA-MB-361 and SKBR111), however, also had levels similar to the non-malignant lines (Figure 4a). We also found FGF-1 product in all of the ten non-mammary cell lines examined, with the A204 cells having the highest levels (similar to those found in the breast tissues) (Figure 4b). Overall, the values normalised to actin showed that expression in the cell lines was considerably lower than that seen in tissue samples.

FGF-1 protein was detectable by immunoblotting in the A204 cells (data not shown) and in the three non-malignant breast cell lines tested (HBrSV1,6.1, HBL100 and MCF10a). A band corresponding to the expected 18 kDa FGF-1 protein was seen in each case. No FGF-1 protein was detected in four of the five breast cancer cell lines tested but a band similar to that seen in the normal cell lines was observed with MDA-MB-231 cancer cells (Figure 5). Immunostaining of these cell lines gave similar results (data not shown) with staining seen only in the non-malignant lines.

In summary, the non-malignant cell lines of both myoepithelial and epithelial origin contain both the FGF-1 mRNA and the translated protein whereas the changes involved in carcinogenesis have led to a reduction in the expression of FGF-1 in 80% of the malignant cell lines tested.

**Figure 5** Western blot analysis of FGF-1 protein in cell lines. Recombinant FGF-1 (10 ng) (lane 1) and extracts from HBL-100 (lane 2), MCF10a (lane 3), MDA-MB-231 (lane 4), MCF7 (lane 5), T47D (lane 6), ZR75-1 (lane 7), SKBR111 (lane 8) and HBRSV1.6.1 (lane 9) cell lines were run on a 7.5% SDS-PAGE, transferred to nitrocellulose and probed with an anti-FGF-1 antibody (British Biotechnology), followed by anti-rabbit IgG -peroxidase conjugate and developed using ECL reagents.

### Discussion

In this study, we have compared the expression of FGF-1 mRNA and protein in malignant and non-malignant breast using 153 tissue samples and 14 breast derived cell lines. Using all methods we noted a decrease in the expression of FGF-1 in breast cancer. Semiquantitative PCR allowed the detection of FGF-1 mRNA in all the tissue samples, however the levels seen in cancers were significantly lower than those seen in non-malignant tissues. This finding is in marked contrast to a study of FGF-1 expression in pancreatic cancer in which FGF-1 mRNA was found to be overexpressed in cancer with expression levels correlating with tumour stage (Yamanaka *et al*., 1993).

We have confirmed our results by using Western blotting to monitor the level of FGF-1 protein present in breast tissues. The same pattern of expression is seen, with non-malignant cell lines and tissue samples containing higher levels of FGF-1 than cancer cell lines and tissue samples. FGF-1 would be expected to be translated as a single form of 18 kDa and this single band is seen in all benign cell lines and tissues analysed (Burgess and Maciag, 1989; Cao and Pettersson, 1993). Additional bands of 14 kDa and 16 kDa are seen in reduction mammoplasty tissue. This process was required because the higher fat content of non-malignant tissue compared with breast tumours made it difficult to achieve lysates of normal tissue with protein concentrations similar to those of the cancer sample lysates without using organoid preparation as a way of enriching for cells.

The cellular localisation of FGF-1 was studied on cryostat sections using immunohistochemistry. FGF-1 protein was found predominantly in the luminal epithelial cells of normal ducts. This is in agreement with a previous study showing strong anti-FGF-1 immunoreactivity in the glandular epithelium (Hughes and Hall, 1993). Again, a large decrease in FGF-1 expression was seen in breast cancer, with no staining apparent in breast cancer cells. Thus luminal epithelial cells normally express FGF-1 and transformation of these cells results in loss of expression of FGF-1.

The study of FGF-1 expression in breast-derived cell lines showed that non-malignant cell lines of both epithelial and myoepithelial phenotypes expressed FGF-1. We observe staining only on the luminal epithelial cells of cryostat sections and two theories could explain this difference. One possibility is that myoepithelial cells do not express FGF-1 under normal conditions in the breast, however changes involved in growing myoepithelial cells in tissue culture conditions might induce expression of FGF-1. Alternatively, both cell types may express FGF-1 in the breast but FGF-1 becomes associated predominantly with the luminal epithelial cells *in vivo*. *In situ* hybridization experiments would be required to assess the situation *in vivo*. Anandappa *et al*. (1994) have reported decreases in FGF-1 mRNA expression in the malignant breast using Northern blot experiments. This less sensitive technique did not allow detection of FGF-1 mRNA in epithelial and myoepithelial cells and stromal elements were suggested as the source of FGF-1. Our studies identify non-malignant epithelial cells as the expressors of FGF-1.

FGF-1 is present in normal breast ducts and we and others have detected receptors for FGF-1 on both epithelial and myoepithelial cells (Luqmani *et al*., 1992; Jacquemier *et al*., 1994; McLeskey *et al*., 1994). This raises the possibility that FGF-1 has roles in autocrine or paracrine control of epithelial and myoepithelial cells in the normal duct. The role of FGF-1 in the normal duct is unclear since, although FGF-1 can stimulate mitogenesis in some breast cancer cell lines (Briozzo *et al*., 1991; Johnston *et al*., 1995), there have been reports of FGF-1 treatment leading to a decreased rate of growth (McLeskey *et al*., 1994). FGF stimulation of PC12 cells leads to differentiation rather than proliferation and it is possible that the function of FGF-1 is to maintain the differentiated state of the duct rather than cause cell proliferation (Kremer *et al*., 1991). Our results show a dramatic decrease in the amount of both FGF-1 mRNA and protein in breast cancer compared with non-malignant biopsy samples. This may lead to the loss of any regulatory function performed in the breast by FGF-1. If FGF-1 has a role in maintaining the differentiated state of the breast duct, then loss of FGF-1 expression may contribute to the malignant phenotype.

An apparently contradictory situation occurs for FGF receptors. Experiments have shown an increase in the number of receptors in breast cancer compared with normal epithelial cells. Gene amplification of the FGFR-4 gene has been found in 10% of breast cancers (Jaakkola *et al*., 1993). Increased expression of FGFR-1 has been found in 15% of breast tumours and a panel of breast cancer cell lines show amplification of either FGFR-1 or FGFR-4 in several cell lines (Jacquemier *et al*., 1994; McLeskey *et al*., 1994). Both of these receptors would bind FGF-1 with high affinity, however in the absence of FGF-1 in breast cancer it is possible that these overexpressed receptors will be activated by an alternative ligand such as FGF-4 or FGF-6, although neither of these has been detected in the breast (Vainikka *et al*., 1992). It has recently been reported that FGF receptors also interact with adhesion molecules, with such interactions leading to receptor activation and $Ca^{2+}$ influx (Williams *et al*., 1994). It is possible that alternative interactions such as these are responsible for stimulating the overexpressed FGF receptors in breast cancer.

The decrease in FGF-1 expression in breast cancer cells is striking. It is a frequent change occurring in carcinogenesis since at least 80% of breast cancers contain no detectable FGF-1 by Western blot analysis and immunohistochemistry. Further investigation will be required to assess the functional effect of the decrease in this growth factor. The presence of FGF-1 and its receptors in the normal breast suggests a role for this factor in the maintenance of the normal duct. The decrease in FGF-1 in cancer would be expected to perturb the epithelial cells and could be influential in the progression of carcinogenesis.

### Abbreviations

FGF-1 and -2, acidic and basic fibroblast growth factor; FGFR, fibroblast growth factor receptor; RT, reverse transcriptase; PCR, polymerase chain reaction.

### Acknowledgements

We are grateful to Jean Walters and Professor Nigel Groome from the Brookes University, Oxford, for help in raising a monoclonal antibody against FGF-1. We are grateful to the Buckle Family Trust for funding CY. This work was supported by the Cancer Research Campaign.

### Note added in proof

Since writing this paper, a more extensive immunocytochemical study of FGF-1 expression in frozen sections of human breast has been carried out. We continue to see a decrease in FAF-1 expression in malignant epithelial cells, however FGF-1 staining is seen in the stroma surrounding malignant epithelial cells, whereas no stromal staining is seen in non-malignant samples. This is discussed fully in a further paper by R Coope *et al.*, submitted to *Cancer Research.*

### References

ABRAHAM J, MERGIA A, WHANG J, TUMULO A, FRIEDMAN J, HJERRILD K, GOSPODAROWICZ D AND FIDDES J. (1986). Nucleotide sequence of a bovine clone encoding the angiogenic protein, basic fibroblast growth factor. *Science*, **233**, 545-548.

ANANDAPPA SY, WINSTANLEY, JHR, LEINSTER S, GREEN B, RUDLAND PS AND BARRACLOUGH R. (1994). Comparative expression of fibroblast growth factor mRNAs in benign and malignant breast disease. *Br. J. Cancer*, **69**, 772-776.

BARRITAULD D, GROUX-MUSCATELLI B, CARUELLE D, VOISIN M-C, CHOPIN D AND PLACY S. (1991). Acidic fibroblast growth factor content increases with malignancy in human chondrosarcoma and bladder cancer. *Ann. NY Acad. Sci.*, **638**, 387-393.

BRADFORD M. (1976). A rapid and sensitive method for the quantitation of microgram quantities of protein utilising the principles of protein dye binding. *Anal. Biochem.*, **72**, 248-256.

BRIOZZO P, BADET J, CAPONY F, PIERI I, MONTCOURRIER P, BARRITAULD D AND ROCHEFORT H. (1991). MCF7 cells respond to bFGF and internalise it following its release from the extracellular matrix; a permissive role for cathepsin D. *Exp. Cell Res.*, **194**, 252-259.

BURGESS W AND MACIAG T. (1989). The heparin-binding (fibroblast) growth factor family of proteins. *Annu. Rev. Biochem.*, **58**, 575-606.

CAO Y AND PETTERSSON R. (1993). Release and subcellular localisation of acidic fibroblast growth factor expressed to high levels in HeLa cells. *J. Cell Science*, **104**, 77-87.

CHAO HH, YANG VC AND CHEN JK. (1993). Acidic FGF and EGF are involved in the autocrine growth stimulation of a human-nasopharyngeal carcinoma cell line and sub-line cells. *Int. J. Cancer*, **54**, 807-812.

CHIRGWIN SM, PRZYBYLA AE, MACDONALD RJ AND RUTTER WJ. (1979). Isolation of biologically active ribonucleic acid from sources enriched in ribonucleases. *Biochemistry*, **18**, 5294-5299.

CHOMCZYNSKI P AND SAACHI N. (1987). Single step method of RNA isolation by acid guanidinium thiocyanate phenol-chloroform extraction. *Anal. Biochem.*, **162**, 156-159.

CHURCH GM AND GILBERT W. (1984). Gemomic sequencing. *Proc. Natl Acad. Sci. USA*, **81**, 1991-1995.

DIONNE C, CRUMLEY G, BELLOT F, KAPLOW J, SEARFOSS G AND RUTA M. (1990). Cloning and expression of two distinct high-affinity receptors cross-reacting with acidic and basic fibroblast growth factors. *EMBO J.*, **9**, 2685-2692.

FEINBERG AP AND VOGELSTEIN B. (1983). A technique for radiolabelling DNA restriction endonuclease fragments to high specific activity. *Anal. Biochem.*, **132**, 6-13.

GOLDFARB M. (1990). The fibroblast growth factor family. *Cell. Growth Diff.*, **1**, 439-445.

GOMM J, SMITH J, RYALL G, BAILLIE R, TURNBULL L AND COOMBES C. (1991). Localisation of basic fibroblast growth factor and transforming growth factor b1 in the human mammary gland. *Cancer Res.*, **51**, 4685-4692.

GOSPODAROWICZ D, NEUFELD G AND SCHEIGER L. (1987). Fibroblast growth factor: structure and biological properties. *J. Cell. Physiol.*, **5**, (suppl.) 15-26.

HOU J, KAN M, MCKEEHAN K, MCBRIDE G, ADAMS P AND MCKEEHAN W. (1991). Fibroblast growth factor receptors from liver vary in three structural domains. *Science*, **251**, 665-668.

HUGHES SE AND HALL PA. (1993). Immunolocalisation of fibroblast growth factor receptor 1 and its ligands in human tissue. *Lab. Invest.*, **69**, 173-182.

JAAKKOLA S, SALMIKANGAS P, NYLUND S, PARTANEN J, ARMSTRONG E, PYRHONEN S, LEHTOVIRTA P AND NEVANLINNA H. (1993). Amplification of FGFR4 gene in human breast and gynecological cancers. *Int. J. Cancer*, **54**, 378-382.

JACQUEMIER J, ADELAIDE J, PARC P, PENAULD-LLORCA F, PLANCHE J, DELAPEYRIERE O AND BIRNBAUM D. (1994). Expression of the FGFR-1 gene in human breast carcinoma cells. *Int. J. Cancer*, **59**, 373-378.

JAYE M, HOWK R, BURGESS W, RICCA G, CHIU I, RAVERA M, O'BRIAN S, MODI W, MACIAG T AND DROHAN W. (1986). Human endothelial growth factor: cloning, nucleotide sequence and chromosome localisation. *Science*, **233**, 541-545.

JAYE M, SCHLESSINGER J AND DIONNE C. (1992). Fibroblast growth factor receptor tyrosine kinases: molecular analysis and signal transduction. *Biochim. Biophys. Acta.*, **1135**, 185-199.

JOHNSON D, LU J, CHEN H, WERNER S AND WILLIAMS L. (1991). The human fibroblast growth factor receptor genes: a common structural arrangement underlies the mechanism for generating receptro forms that differ in their third immunoglobulin domain. *Mol. Cell. Biol.*, **11**, 4627-4634.

JOHNSTON CL, COX H, GOMM JJ AND COOMBES RC. (1995). bFGF and aFGF induce membrane ruffling in breast cancer cells but not in normal breast epithelial cells: FGFR-4 involvement. *Biochem. J.*, **306**, 609-616.

KAN M, WANG F, XU J, CRABB J, HOU J AND MCKEEHAN W. (1993). An essential heparin-binding domain in the fibroblast growth factor receptor kinase. *Science*, **259**, 1918-1921.

KEEGAN K, JOHNSON D, WILLIAMS L AND HAYMAN M. (1991). Isolation of an additional member of the fibroblast growth factor receptor family. *Proc. Natl Acad. Sci. USA.*, **88**, 1095-1099.

KHAN I, FISHER RA, JOHNSON KJ, BAILEY M, SCILIANO MJ, KESSLING A, FARRER M, CARRIT B, KAMALATI T AND BULUWELA L. (1994). The SON gene encodes a conserved DNA binding proteins mapping to human chromosome 21. *Hum. Genet.*, **58**, 25-34.

KLAGSBRUN M. (1990). The affinity of fibroblast growth factors (FGFs) for heparin: FGF-heparin sulphate interactions in cells and extracellular matrix. *Curr. Opin. Cell. Biol.*, **2**, 857-863.

KLAGSBRUN M AND BAIRD A. (1991). A dual receptor system is required for basic fibroblast growth factor activity. *Cell*, **67**, 229-231.

KORNBLUTH S, PAULSON K AND HANAFUSA H. (1988). Novel tyrosine kinase identified by phosphotyrosine antibody screening of cDNA libraries. *Mol. Cell. Biol.*, **8**, 5541-5544.

KREMER NE, D'ARCANGELO G, THOMAS SM, DEMARCO M, BRUGGE JS AND HALEGOUA S. (1991). Signal transduction by nerve growth factor and fibroblast growth factor in PC12 cells requires a sequence of ras and src actions. *J. Cell. Biol.*, **115**, 809-819.

LEE P, JOHNSON D, COUSENS L, FRIED V AND WILLIAMS L. (1989). Purification and cDNA cloning of a receptor for basic fibroblast growth factor. *Science*, **245**, 57-59.

LUQMANI YA, GRAHAM M AND COOMBES RC. (1992). Expression of basic fibroblast growth factor, FGFR-1 and FGFR-2 in normal and malignant human breast and comparison with other normal tissues. *Br. J. Cancer*, **66**, 273-280.

MCLESKEY SW, DING I, LIPPMAN M AND KERN FG. (1994). MDA-MB-134 breast carcinoma cells overexpress fibroblast growth factor receptors and are growth inhibited by FGF ligands. *Cancer Res.*, **54**, 523-530.

MANSUKHANI A, DELL'ERA, P, MOSCATELLI D, KORNBLUTH S, HANAFUSA H AND BASILICO C. (1992). Characterization of the murine BEK fibroblast growth factor (FGF) receptor: Activation by three members of the FGF family and requirement for heparin. *Proc. Natl Acad. Sci. USA.*, **89**, 3305-3309.

MIGNATTI P, MORIMOTO T AND RIFKIN D. (1992). Basic fibroblast growth factor, a protein devoid of secretary signal sequence, is released from cells via a pathway independeny of the endoplasmic reticulum-Golgi complex. *J. Cell. Physiol.*, **151**, 81-93.

MIKI T, BOTTARO D, FLEMING T, SMITH C, BURGESS W, CHAN A AND AARONSON S. (1992). Determination of ligand-binding specificity by alternative splicing: Two distinct growth factor receptors encoded by a single gene. *Proc. Natl Acad. Sci. USA.*, **89**, 246-250.

ORNITZ D, YAYON A, FLANAGAN J, SVAHN C, LEVI E AND LEDER P. (1992). Heparin is required for cell-free binding of basic fibroblast growth factor to a soluble receptor for mitogenesis in whole cells. *Mol. Cell. Biol.*, **12**, 240-247.

PARTANEN J, MAKELA T, EEROLA E, KORHONM J, HIRVONEN H, CLAESSON-WELSH L AND ALITALO K. (1991). FGFR-4, a novel acidic fibroblast growth factor receptor with a distinct expression pattern. *EMBO J.*, **10**, 1347-1354.

RAPRAEGER A, KRUFKA A AND OLWIN B. (1991). Requirement of heparin sulphate for bFGF-mediated fibroblast growth and myoblast differentiation. *Science*, **252**, 1705-1707.

RON D, REICH R, CHEDID M, LENGEL C, COHEN O, CHAN A, NEUFELD G, MIKI T AND TRONICK S. (1993). Fibroblast growth factor receptor 4 is a high affinity receptor for both acidic and basic fibroblast growth factor but not for kerinocyte growth factor. *J. Biol. Chem.*, **268**, 5388-5394.

SMITH J, YELLAND A, BAILLIE R AND COOMBES RC. (1994). Acidic and basic fibroblast growth factors in human breast tissue. *Eur. J. Cancer*, **30A**, 496-503.

STAMPFER M, HALLOWES RC AND HACKETT AJ. (1980). Growth of normal human mammary cells in culture. *in vitro*, **16**, 415-425.

VAINIKKA S, PARTANEN J, BELLOSTA P, COULIER F, BASILICO C, JAYE M AND ALITALO K. (1992). Fibroblast growth factor receptor 4 shows novel features in genomic structure, ligand binding and signal transduction. *EMBO J.*, **11**, 4273-4280.

VIJAYAN VK, LEE YL AND ENG LF. (1993). Immunohistochemical localisation of basic fibroblast growth factor in cultivated rat astrocytes and oligodendrocytes. *Int. J. Dev. Neurosci.*, **11**, 257-267.

WILLIAMS EJ, FURNESS J, WASH FS AND DOHERTY P. (1994). Activation of the FGF receptor underlines neurite outgrowth stimulated by L1, N-CAM and N-Cadherin. *Neuron*, **13**, 583-594.

YAMANAKA Y, FRIESS H, BUCHLER M, BEGER HG, UCHIDA E, ONDA M, KOBRIN MS AND KORC M. (1993). Overexpression of acidic and basic fibroblast growth factors in human pancreatic cancer correlates with advanced tumour stage. *Cancer Res.*, **53**, 5289-5296.

YAYON A, KLAGSBRUN M, ESKO J, LEDER P AND ORNITZ D. (1991). Cell surface, heparin-like molecules are required for binding of basic fibroblast growth factor to its high affinity receptor. *Cell*, **64**, 841-848.

YAYON A, ZIMMER Y, GUO-HONG S, AVIVI A, YARDEN Y AND GIVOL D. (1992). A confined variable region confers ligand specificity on fibroblast growth factor receptors: implication for the origin of the immunoglobulin fold. *EMBO J.*, **11**, 1885-1890.